# Low-Light Image Enhancement Based on Multi-Path Interaction

**DOI:** 10.3390/s21154986

**Published:** 2021-07-22

**Authors:** Bai Zhao, Xiaolin Gong, Jian Wang, Lingchao Zhao

**Affiliations:** 1 School of Microelectronics, Tianjin University, Tianjin 300072, China; zhaobai@tju.edu.cn (B.Z.); zhaolingchao@tju.edu.cn (L.Z.); 2 School of Electrical and Information Engineering, Tianjin University, Tianjin 300072, China; jianwang@tju.edu.cn; 3 National Ocean Technology Center, Tianjin 300112, China

**Keywords:** low-light image, color channel, multi-path interaction, convolutional neural network

## Abstract

Due to the non-uniform illumination conditions, images captured by sensors often suffer from uneven brightness, low contrast and noise. In order to improve the quality of the image, in this paper, a multi-path interaction network is proposed to enhance the R, G, B channels, and then the three channels are combined into the color image and further adjusted in detail. In the multi-path interaction network, the feature maps in several encoding–decoding subnetworks are used to exchange information across paths, while a high-resolution path is retained to enrich the feature representation. Meanwhile, in order to avoid the possible unnatural results caused by the separation of the R, G, B channels, the output of the multi-path interaction network is corrected in detail to obtain the final enhancement results. Experimental results show that the proposed method can effectively improve the visual quality of low-light images, and the performance is better than the state-of-the-art methods.

## 1. Introduction

With the development of computer technology and camera sensors, computer vision has been applied in various engineering fields—for example, object detection in autonomous vehicles [1] and harvesting robots [2], detection and monitoring in the field of civil engineering [3,4], video surveillance [5], 3D reconstruction [6] and so on. Since vision tasks play an important role in a wide range of fields, reliable working performance is required. However, the tasks rely on scene illumination, and the performance of any camera-sensor-based perception tasks is highly degraded in poor illumination conditions such as low-light scenes [7]. In low-light scenes, when a camera cannot receive sufficient light or the camera sensor is not sufficiently sensitive, the captured images may have problems such as poor visualization and low image quality, making the valid information of the image disturbed and limiting the use of the image in computer vision tasks [8,9]. As we know, the degradation of the low-light images captured in a non-uniform illumination environment results in severe object information loss and makes the object detection more challenging [10]. The camera’s night mode sometimes suppresses this degradation; however, a slight shake may introduce other problems such as blurring. Improving the illumination of the environment or updating the camera sensor is not feasible in some conditions [11]. Therefore, low-light image enhancement methods at the software end are needed.

At present, a large number of image enhancement methods have been proposed. Histogram equalization (HE)-based methods [12,13] redistribute pixel values according to the cumulative distribution function of the input image to expand the dynamic range. For example, Ibrahim et al. [14] smoothed the input histogram with a one-dimensional Gaussian filter, and then partitioned the smoothed histogram based on its local maximums. After each partition was assigned to a new dynamic range, the histogram equalization process was applied independently to these partitions. The last step in this method was to normalize the output image to the input mean brightness. Ying et al. [15] and Ren et al. [16] utilized the input image and camera response model to adjust the pixel values. The methods based on Retinex theory [17] adaptively adjusted the illuminance and reflectance components of the image, where the reflectance component was considered as an inherent attribute of the scene and was unchangeable in different lighting conditions [18,19]. Jobson et al. [20] extended a previously designed single-scale center/surround Retinex to a multiscale version that achieved simultaneous dynamic range compression/color consistency/lightness rendition. In order to correct the deficiency present in the extension, a method of color restoration, at the cost of a modest dilution in color consistency, was defined. Fu et al. [21] derived two inputs that represented luminance-improved and contrast-enhanced versions of the decomposed illumination using the sigmoid function and adaptive histogram equalization, and then fused the derived inputs with the corresponding weights in a multiscale fashion to adjust illumination. The method combined the advantages of sigmoid function and histogram equalization, and the final enhanced image was obtained by compensating the adjusted illumination back to the reflectance. Dong et al. [22] noticed that the inverted low-light images intuitively resembled images acquired in hazy lighting conditions; thus, low-lighting image enhancement has much in common with video haze removal. Therefore, they applied image de-hazing algorithm to the inverted image to enhance the image. These methods are simple and effective. However, the results may have undesirable illumination and amplified noise.

In recent years, with the improvement of computer performance and the establishment of publicly available datasets, image enhancement methods based on convolutional neural networks (CNNs) have been actively researched. The CNN-based method is one of the data-driven methods and uses paired images for end-to-end learning. Wei et al. [23] proposed a Retinex-Net learned on a real dataset, which includes a Decom-Net to decompose low-light images into illumination and reflectance components and an Enhance-Net to adjust the illumination component. Xu et al. [24] observed that noise exhibits different levels of contrast in different frequency layers, and it is much easier to detect noise in the low-frequency layer than in the high-frequency one. Therefore, they proposed a network that learns to recover image objects in the low-frequency layer and then enhances high-frequency details based on the recovered image objects. Chen et al. [25] used an exposure prediction network to generate under-/overexposure images and then fused them with the input image to obtain the enhanced image. Lv et al. [26] proposed a multi-branch network to extract rich features of different levels and then fused the multi-branch outputs to produce the output image. Wang et al. [27] considered the low-light image enhancement as a residual learning problem. They proposed a deep lightening network, which consists of several lightening back-projection blocks that perform lightening and darkening processes iteratively to learn the residual for normal-light estimations. Moreover, a feature aggregation block that adaptively fuses the results of different lightening back-projection blocks was designed to effectively utilize the local and global features. Ma et al. [11] transformed the original low-light image from the RGB to HSI color space and used the segmentation exponential method to process the saturation (S) while applying a specially designed deep convolutional neural network to enhance the intensity component (I). The final improved image could be obtained by going back to the original RGB space. Lore et al. [28] used a class of deep neural networks, a stacked sparse denoising autoencoder (SSDA), to enhance natural low-light images. They explored two types of deep architecture, including learning contrast-enhancement and denoising simultaneously, and learning contrast-enhancement and denoising sequentially. CNN-based methods are effective in preserving details and denoising. Nevertheless, existing methods may not perform well on color.

Another data-driven method is the generative adversarial network (GAN)-based method. Different from CNN-based methods, GAN-based methods do not require strictly paired images. They usually require careful selection of unpaired training data. Each GAN contains a generator to output enhanced images and a discriminator to determine whether the output produced by the generator is satisfactory. Jiang et al. [29] proposed to regularize the unpaired training using the information extracted from the input itself, and used a global-local discriminator structure to handle spatially varying light conditions in the input image, while adding the idea of self-regularization, which is implemented by both the self feature preserving loss and the self-regularized attention mechanism. Chen et al. [30] augmented the U-Net with global features and improved Wasserstein GAN (WGAN) with an adaptive weighting scheme, then used individual batch normalization layers for generators in two-way GANs to help generators better adapt to their own input distributions. The design improves the stability of GAN training for the application. Liu et al. [31] proposed a perceptual-details GAN utilizing ZeroDCE to initially recover illumination and combined a residual dense-block encoder–decoder structure to suppress noise while finely adjusting the illumination. In addition, the details were enhanced by using fractional differential gradient masks integrated into the discriminator. However, the generator may collapse due to the fact that the discriminator fails to discriminate its output, and it is difficult to obtain the desired output from two models with opposite objectives trained simultaneously [27].

In order to effectively enhance the brightness of low-light images while restoring the color and details, we propose an end-to-end learning method. The method consists of two cascaded subnetworks that first enhance the color channels and then adjust the details to obtain enhanced images with good color restoration. The enhanced images are expected to display improved visual quality and enhanced performance in computer vision tasks such as object detection and instance segmentation [32,33], and an example of text recognition is shown in Section 3.4. Overall, our contributions are as follows:

(1) The low-light image enhancement task is simplified into three steps. The first step is the enhancement of R, G, and B channels; then, the reconstruction of the color image is performed, and the last step is the adjustment of details.

(2) We design a multi-path interaction network (MPI-net) to enhance the R, G, and B channels. Then, through the interaction across the parallel paths, the feature maps are potentially more accurate.

(3) With the help of exposure amplification loss in the detail correction network (DC-net) and other losses, the final enhanced images are more natural. The experimental results demonstrate that our method outperforms several state-of-the-art enhancement methods.

## 2. Proposed Method

Networks inspired by U-Net [34] are often a single path from high-resolution to low-resolution for encoding, and low-resolution to high-resolution for decoding, where usually only the skip connections directly concatenate the feature maps in the downsampling layer to its corresponding upsampling layer according to space resolution to increase the amount of information in the upsampling steps [35]. In order to increase the information representation of feature maps in the network, we design a multi-path interaction network (MPI-net), which extends the network structure based on the U-Net idea to further enhance the information interaction between feature maps of different resolutions, while retaining a high-resolution path and enhancing the utilization of information in the network. Retaining the high-resolution path in the network, rather than upsampling to high-resolution from low-resolution, potentially leads to more accurate feature maps [36].

**Figure 1:**
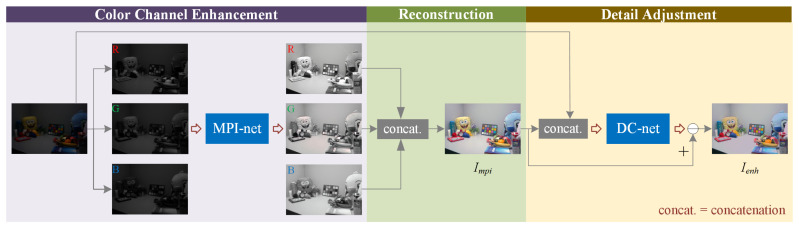
The architecture of the proposed method. The enhancement process is divided into three steps: color channel enhancement, reconstruction, detail adjustment. In the color channel enhancement step, a subnetwork MPI-net enhances the R, G, B channels. In the reconstruction step, the enhanced R, G, B channels are concatenated in order to generate the preliminary enhanced image $I_{mpi}$. In the detail adjustment step, we concatenate the $I_{mpi}$ and the input image as the input of DC-net, and the output of DC-net is a residual map. The final enhanced image $I_{enh}$ is obtained by subtracting the residual map from the $I_{mpi}$.

We consider the image enhancement as the enhancement of three channels. At first, the R, G, and B channels of low-light images are trained separately to obtain the enhanced R, G, and B channels and recombine them into a color image. Since the three channels are trained separately, the correlation between the color channels is ignored and the obtained images may have unnatural colors and overexposure. Therefore, a detail correction network (DC-net) is used after the multi-path interaction network (MPI-net) to further adjust the color images generated by the output of MPI-net. The DC-net consists of several convolutions, and the last layer of the convolution is a residual map. The enhanced images can be obtained by subtracting the residual maps from the color images generated by the output of MPI-net. The overall architecture of the proposed method is shown in Figure 1.

### 2.1. Multi-Path Interaction Network

#### 2.1.1. Network

**Figure 2:**
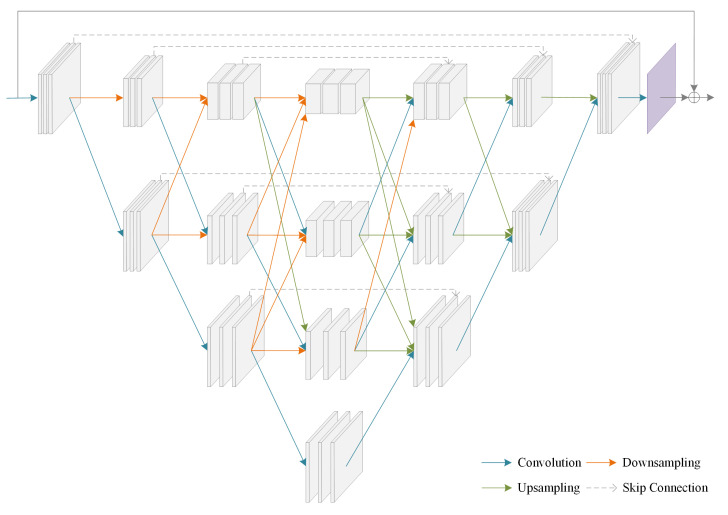
The architecture of MPI-net.

The first path of the multi-path interaction network (MPI-net) is a high-to-low and low-to-high resolution network (HL-net), and the number of HL-Net paths is gradually increased one by one to form more paths until the last path contains only high-resolution feature maps. The paths are connected in parallel, and the feature maps for the parallel paths of a later stage consist of the feature maps from the previous stage and an extra lower one. Meanwhile, there is a high-resolution path in the network. The architecture of MPI-net is shown in Figure 2. The MPI-net connects multiple paths to form a richer feature representation while retaining the ability of U-Net. At the same time, the existence of a high-resolution path and the interaction of information between feature maps of the same or different resolutions in different paths make the feature representation potentially more accurate [36]. In one path, each downsampling step is a convolution with stride 2. Each upsampling step contains a bilinear interpolation to expand the size of the feature map to twice the original. Moreover, three cascaded convolutional layers are included between two operations with different spatial resolutions. Each convolutional layer consists of a 3 × 3 convolution operation with padding, followed by a rectified linear unit (ReLU) activation function. In addition, skip connections directly concatenate the feature maps in the downsampling layer to the corresponding upsampling layer to increase the amount of information in the upsampling steps. The number of channels of feature maps with different resolutions in the first path is 32, 64, 128 and 256, respectively. Moreover, other paths are consistent with the first path in the number of feature map channels.

**Figure 3:**
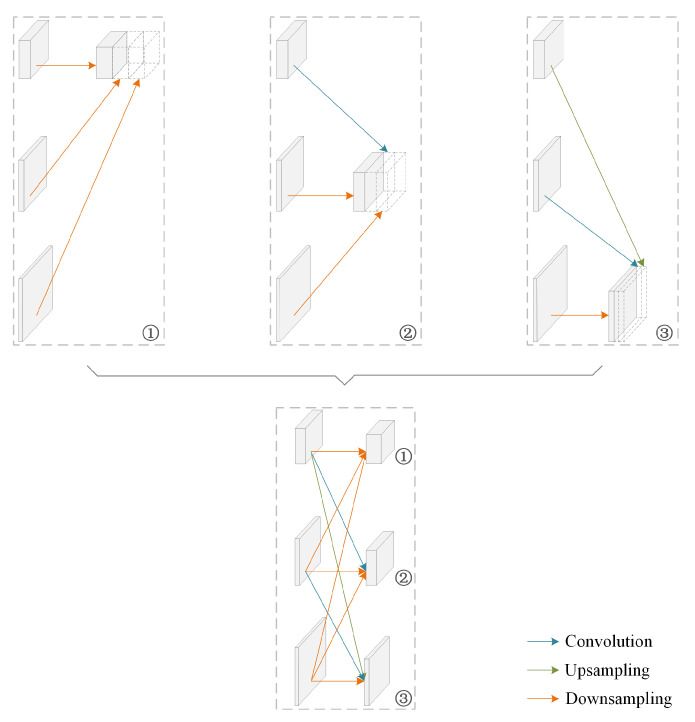
Illustrating how the exchange unit aggregates the information for different paths. Feature maps from different paths are transformed to the same resolution and then concatenated.

The exchange of information between feature maps of different resolutions leads to rich resolution representations [36]. Therefore, the exchange units are introduced across parallel paths in the MPI-net. An example is shown in Figure 3. Since the paths are connected in parallel, each path repeatedly receives the information from the other parallel paths. The feature maps for exchanging information are at the same depth in the network and usually have different resolutions. In the exchange unit, for different paths, they are transformed to the same resolution and concatenated on the path to complete the information exchange. Both upsampling and downsampling are used only once in one exchange unit.

#### 2.1.2. Loss Function

The loss function of MPI-net consists of two components, the mean square error loss $L_{mse}$ and the structural similarity loss $L_{ssim}$, expressed as follows:

(1) $$L_{mpi-net}=L_{mse}^{mpi-net}+\lambda_{1}L_{ssim}^{mpi-net}$$

where λ is used to control the image structure.

The mean squared error (MSE) is the average of the squared sum of the corresponding pixel errors between the enhanced channel and the reference channel, and is used to evaluate the overall difference between two channels. A smaller MSE means a better result. Therefore, the mean square error loss $L_{mse}^{mpi-net}$ is defined as:

(2) $$L_{mse}^{mpi-net}=\frac{1}{H\times W}\sum_{c\in \{R,G,B\}}\left|\right|I_{mpi\_c}-I_{ref\_c}{\left|\right|}_{2}^{2}$$

where $I_{mpi\_c}$ is the enhanced c channel, $I_{ref\_c}$ is the c channel of the reference image, $\left|\right|\cdot {\left|\right|}_{2}$ means $L_{2}$ norm, *H* and *W* are the height and width of the image.

The structural similarity (SSIM; [37]) is used to evaluate the similarity of two channels in terms of luminance, contrast and structure. The value of SSIM ranges from 0 to 1, and a larger value indicates better similarity. The definition of SSIM is as follows:

(3) $$SSIM\left(mpi\_c,ref\_c\right)=\frac{\left(2\mu_{mpi\_c}\mu_{ref\_c}+C_{1}\right)\left(2\sigma_{mpi\_c,ref\_c}+C_{2}\right)}{\left(\mu_{mpi\_c}^{2}\mu_{ref\_c}^{2}+C_{1}\right)\left(\sigma_{mpi\_c}^{2}+\sigma_{ref\_c}^{2}+C_{2}\right)}$$

where the parameters *mpi_c* and *ref_c* are simple representations of the enhanced c channel and the reference c channel, $\mu_{mpi\_c}$ is the mean of the $I_{mpi\_c}$, $\mu_{ref\_c}$ is the mean of the $I_{ref\_c}$, $\sigma_{mpi\_c}$ is the variance of the $I_{mpi\_c}$, $\sigma_{ref\_c}$ is the variance of the $I_{ref\_c}$, $\sigma_{mpi\_c,ref\_c}$ is the covariance of the $I_{mid\_c}$ and the $I_{ref\_c}$, $C_{1}$ and $C_{2}$ are constants and take the default values. In order to improve the structural distortion problems that usually exist in low-light images [26], we introduce structural similarity loss $L_{ssim}^{mpi-net}$:

(4) $$L_{ssim}^{mpi-net}=\sum_{c\in \{R,G,B\}}\left(1-SSIM\left(mpi\_c,ref\_c\right)\right)$$

### 2.2. Detail Correction Network

#### 2.2.1. Network

**Figure 4:**
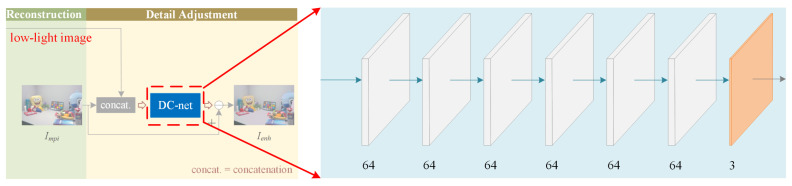
The architecture of the DC-net. The input is the concatenation of the $L_{mpi}$ and the low-light image, and the output is a residual map with detailed information.

The enhanced R, G, B channels are concatenated to generate the preliminary enhanced image ($I_{mpi}$). In order to avoid the loss of details caused by enhancing the color channels separately, the preliminary enhanced image and the low-light image are concatenated as the input of the detail correction network (DC-net) to adjust the details. As shown in Figure 4, the DC-net contains six 64-channel convolution layers, and the 3-channel feature map obtained from the last layer of convolution is a residual map. The final enhanced image is obtained by subtracting the residual map from the preliminary enhanced image. The activation function of the last convolutional layers in MPI-net and DC-net is none.

#### 2.2.2. Loss Function

We introduce DC-net and design extra exposure amplification loss $L_{ea}$ and smoothing loss $L_{smooth}$ to suppress overexposure and make the enhanced images more natural. The total loss function of DC-net is expressed as:

(5) $$L_{dc-net}=L_{mse}^{dc-net}+L_{ssim}^{dc-net}+\lambda_{2}L_{ea}+\lambda_{3}L_{smooth}$$

where $\lambda_{2}$ and $\lambda_{3}$ are used to control the degree of overexposure suppression and smoothing, respectively.

The $L_{mse}^{dc-net}$ is expressed as:

(6) $$L_{mse}^{dc-net}=\frac{1}{H\times W}\left|\right|I_{enh}-I_{ref}{\left|\right|}_{2}^{2}$$

where $I_{enh}$ is the final enhanced image and obtained by subtracting the residual map $I_{res}$ of DC-net from the preliminary enhanced image $I_{mpi}$, and $I_{ref}$ is the reference image.

The $L_{ssim}^{dc-net}$ is expressed as:

(7) $$L_{ssim}^{dc-net}=1-SSIM\left(enh,ref\right)$$

where the parameters *enh* and *ref* are simple representations of the $I_{enh}$ and the $I_{ref}$.

Through the gamma transformation, the pixel difference between the bright areas of the enhanced image and the reference image could be greatly increased, while the pixel difference between the dark areas is slightly increased. Therefore, using the average of the pixel difference between the gamma-transformed enhanced image and the reference image as the loss function helps to place more emphasis on the bright area and suppress overexposure. The exposure amplification loss $L_{ea}$ is defined as follows:

(8) $$L_{ea}=\frac{1}{H\times W}\left|\right|{\left(I_{enh}\right)}^{\gamma }-{\left(I_{ref}\right)}^{\gamma }{\left|\right|}_{1}$$

where $\left|\right|\cdot {\left|\right|}_{1}$ means $L_{1}$ norm, and γ is used to control the increase in the relative difference.

To smooth the enhanced image and make it more natural, we introduce the smoothing loss $L_{smooth}$ to minimize the difference between the horizontal and vertical gradients of the enhanced image and the reference image in the color channels. The definition of smoothing loss $L_{smooth}$ is shown below:

(9) $$L_{smooth}=\frac{1}{H\times W}\sum_{c\in \{R,G,B\}}(||\nabla I_{enhx}^{c}-\nabla I_{refx}^{c}{\left|\right|}_{2}^{2}+||\nabla I_{enhy}^{c}-\nabla I_{refy}^{c}{\left|\right|}_{2}^{2})$$

where $\nabla I_{enhx}^{c}$ is the horizontal gradient of the enhanced image $I_{enh}$ in channel *c*, $\nabla I_{refx}^{c}$ is the horizontal gradient of the reference image $I_{ref}$ in channel *c*, $\nabla I_{enhy}^{c}$ is the vertical gradient of the enhanced image $I_{enh}$ in channel *c*, $\nabla I_{refy}^{c}$ is the vertical gradient of the reference image $I_{ref}$ in channel *c*.

**Figure 5:**
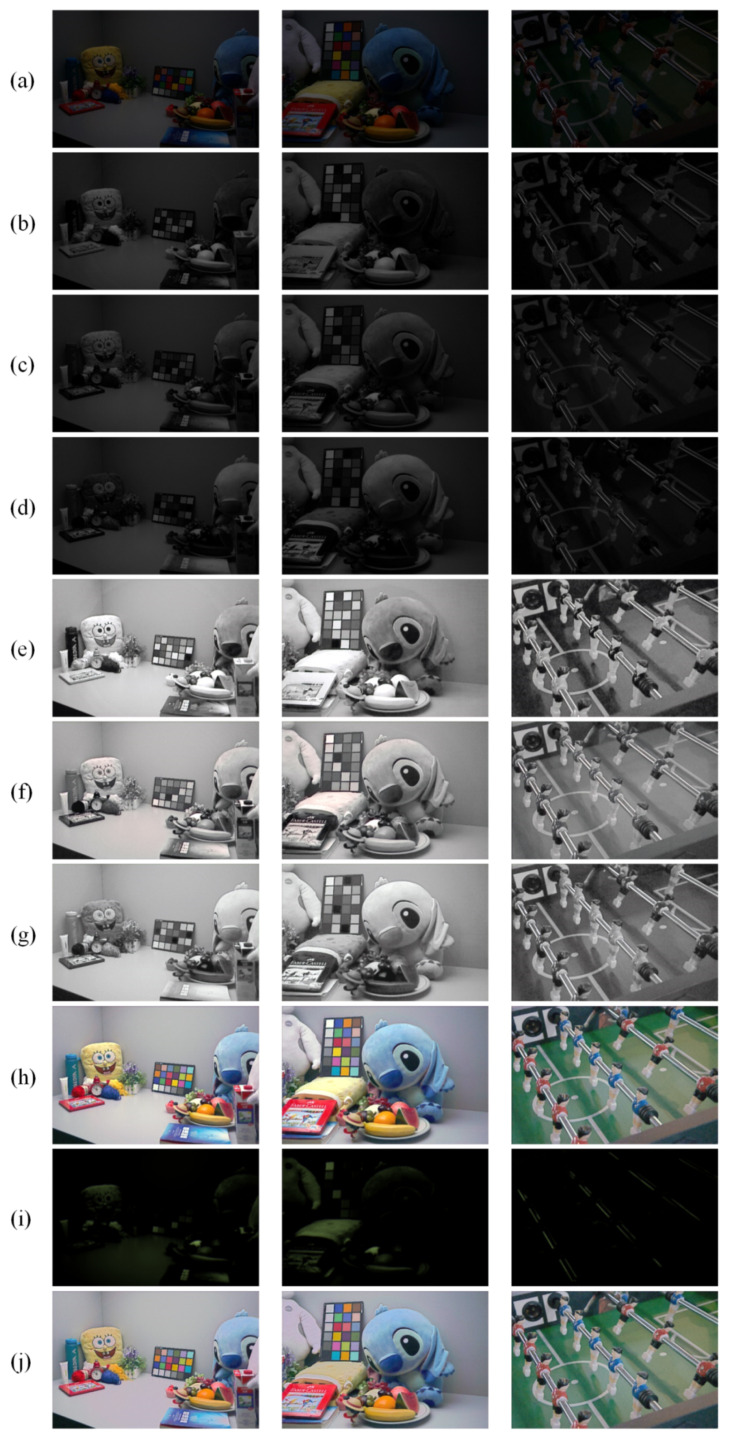
The example of the images used and generated in the proposed method. (**a**): the input images; (**b**–**d**): R, G, and B channels of the input images; (**e**–**g**): the enhanced R, G, and B channels; (**h**): the preliminary enhanced images $I_{mpi}$; (**i**): the residual maps $I_{res}$; (**j**) the final enhanced images $I_{enh}$.

Figure 5 shows an example of the images used and generated in the proposed method, including the input images and its *R*, *G*, and *B* channels, the *R*, *G*, and *B* channels enhanced by the MPI-net and the preliminary enhanced images $I_{mpi}$, the residual maps $I_{res}$ generated by the DC-net, and the obtained enhanced images $I_{enh}$.

## 3. Experiments

### 3.1. Training Details and Dataset

The experiments were carried out using Tensorflow 1.14.0, on a workstation with Intel(R) Xeon(R) CPU E5-2186 @ 3.80 GHz, Nvidia GeForce GTX 2080TI and 64 G RAM. The parameters $\lambda_{1}$, $\lambda_{2}$, $\lambda_{3}$, and γ were set to 3, 1.3, 5, and 5 experimentally. The training images were normalized to [0, 1] and randomly cropped to patches with size of 48×48. The Adam optimizer was used with default parameters and the training epochs for the two subnetworks were both set to 30. For the learning rate, we first initialized it to 0.001 and reduced it by 10 times every 10 epochs. The training could be completed within 5 min.

The training dataset was from the LOL dataset, which is a real dataset containing a training dataset with 485 image pairs and a testing dataset with 15 image pairs. The scenes in the dataset are rich, and the image resolution is 600×400. We selected 234 image pairs of different scenes from the training dataset of the LOL-dataset as the new training dataset, and used the 15 images from the testing dataset of the LOL dataset and another 8 images from the LOL dataset (outside our training dataset) and SICE [38] as the new testing dataset. In addition, images from the LIME [19,39] were also selected to further demonstrate the effectiveness of the proposed model.

### 3.2. Comparison with State-of-the-Art Methods

We compared the proposed method with state-of-the-art methods: BIMEF [15], LECA RM [16], MSRCR [20], LIME [19], MF [21], and Retinex-Net [23]. In addition to analyzing the visual quality of the experimental results, we also adopted peak signal-to-noise ratio (PSNR), structural similarity (SSIM), and natural image quality evaluator (NIQE) [40] to evaluate the image quality.

#### 3.2.1. Visual Quality Comparison

**Figure 6:**
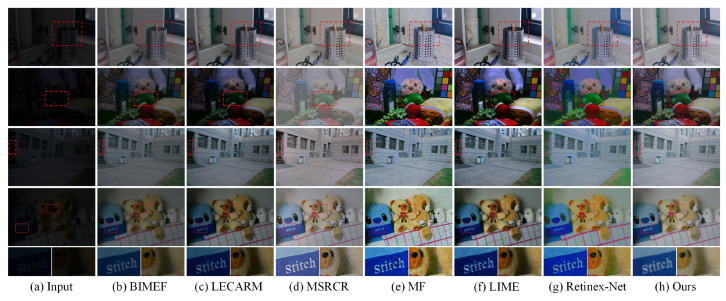
Visual comparison of the proposed method and the state-of-the-art methods on images from LOL dataset but outside our training dataset.

**Figure 7:**
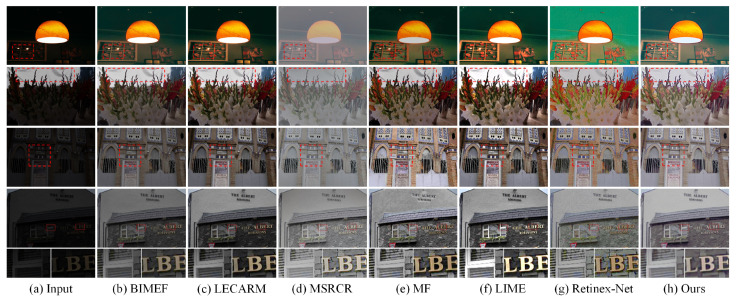
Visual comparison of the proposed method and the state-of-the-art methods on images from LIME [19,39] and SICE [38].

We performed experiments on images with different lighting conditions. The images were from the LOL dataset (outside our training dataset), LIME, and SICE [38,39], and the results are shown in Figure 6 and Figure 7. As shown in Figure 6, although some methods such as LIME have good brightness in local areas, they amplify the noise at the same time. More details can be seen in the last row of Figure 6, where the enhanced images of the proposed method are smooth and noise-free. The brightness of the images enhanced by BIMEF and LECARM is insufficient. The MSRCR has unsatisfactory performance in the contrast of images. In Figure 7, the results of LIME are unnatural in bright areas. Over-enhancement of the input image distorts the color of MF and Retinex-Net. Relatively speaking, the proposed method effectively enhances the brightness and the enhanced images are the most natural.

#### 3.2.2. Evaluation

For a fair comparison, we use the representative metrics PSNR (peak signal-to-noise ratio), SSIM (structural similarity), and NIQE (natural image quality evaluator) to evaluate the image quality of the enhanced images. The PSNR can detect whether an image is distorted. The SSIM measures image similarity from the three aspects of luminance, contrast, and structure. The NIQE is a non-reference image quality evaluation method. We use the average of the images in the testing dataset as the test value and the results are shown in Table 1. The larger the value of PSNR and SSIM, and the smaller the value of NIQE, the better the results. The best results of PSNR, SSIM, and NIQE are bolded in this paper. It can be seen that the proposed method outperforms the other methods in all three metrics. Compared to the best results of the state-of-the-art methods, the proposed method offers a 12.682% improvement in PSNR (compared to LIME), 22.930% improvement in SSIM (compared to BIMEF), and 27.701% improvement in NIQE (compared to BIMEF). This means that the images obtained by the proposed method have the best quality.

### 3.3. Ablation Study

**Figure 8:**
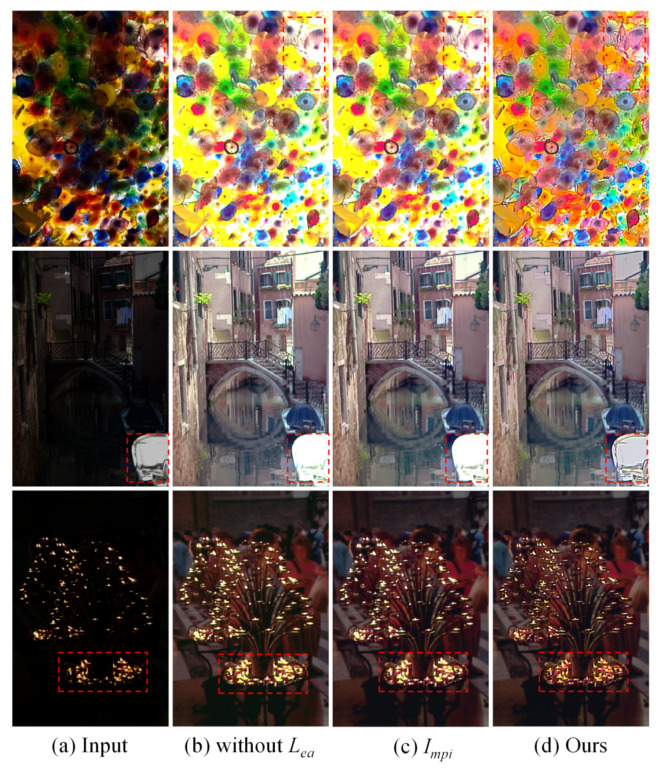
Comparison results of ablation study.

To demonstrate the effectiveness of MPI-net, DC-net, and loss function $L_{ea}$, we conducted an ablation study and analyzed the experimental results. Specifically, we designed experiments: (a) removing the loss function $L_{ea}$; (b) comparing with the preliminary enhanced image $I_{mpi}$. The visual comparison results are presented in Figure 8. As can be seen, without the loss function $L_{ea}$, the bright area is easily overexposed, resulting in a loss of content. Moreover, the preliminary enhanced images $I_{mpi}$ have unsatisfactory performance in bright areas and details. However, the proposed method enhances the dark areas while suppressing overexposure, and the details are natural.

Table 2 illustrates the comparison results in PSNR, SSIM, and NIQE values. We can find that the use of loss function $L_{ea}$ could effectively improve the quality of the enhanced image and the DC-net is necessary. As the color channels are first enhanced separately, sometimes, the DC-net may not perform well and bring color distortion. Nevertheless, the proposed method has satisfactory performance in most scenarios.

### 3.4. Application

**Figure 9:**
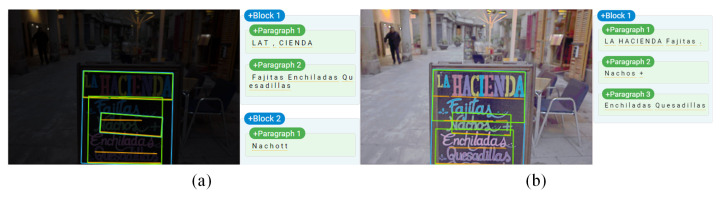
Results of Google Cloud Vision API. (**a**) Recognition result of low-light image; (**b**) Recognition result of our enhanced image.

To further illustrate the effectiveness of the proposed method in improving the accuracy of the computer vision task, we tested our output on Google Vision API (https://cloud.google.com/vision/, accessed on 12 June 2021). The results are shown in Figure 9. As can be seen, the Google Vision API could accurately recognize the text from the enhanced image while recognizing errors when using low-light images. The original image is from SICE [38].

## 4. Conclusions

In this paper, a multi-path interaction network (MPI-net) is designed to enhance the R, G, and B channels separately, and then a detail correction network (DC-net) and corresponding loss functions are used to adjust the details. Thanks to the information interaction between different paths in the MPI-net, the feature maps are potentially more accurate. Moreover, the enhanced images are more natural after the adjustment of DC-net. We compare our method with the state-of-the-art methods, and the experimental results show that the proposed method has better performance. The evaluation metrics of the proposed method are also superior to the state-of-the-art methods. With the wide application of computer vision, it is becoming increasingly important to improve the performance of computer vision tasks in low-light conditions. Our future work will focus on improving the generalization ability of the enhancement model and the enhancement effect in extreme environments, as well as building a complete enhancement and object detection system for nighttime autonomous driving and video surveillance.

## Figures and Tables

**Table 1:** Comparison of BIMEF, LECARM, MSRCR, MF, LIME, Retinex-Net and ours in PSNR, SSIM, and NIQE.

Methods	BIMEF	LECARM	MSRCR	MF	LIME	Retinex-Net	Ours
PSNR	14.050	15.354	15.692	17.103	17.402	17.177	**19.609**
SSIM	0.628	0.598	0.596	0.541	0.575	0.508	**0.772**
NIQE	12.0127	12.8758	13.0186	14.0127	13.2720	15.8324	**8.6851**

**Table 2:** Comparison of performance metrics for ablation study.

Methods	Without $L_{\mathrm{ea}}$	$I_{\mathrm{mpi}}$	Ours
PSNR	19.145	19.321	**19.609**
SSIM	0.768	0.770	**0.772**
NIQE	9.1392	8.8136	**8.6851**
